# Semaphorin 3 C is a Novel Adipokine Representing Exercise-Induced Improvements of Metabolism in Metabolically Healthy Obese Young Males

**DOI:** 10.1038/s41598-020-67004-7

**Published:** 2020-06-19

**Authors:** Ji Sun Nam, Chul Woo Ahn, Hye Jun Park, Yu Sik Kim

**Affiliations:** 1 Department of Internal Medicine, Gangnam Severance Hospital, Yonsei University College of Medicine, Seoul, Korea; 2 Severance Institute for Vascular and Metabolic Research, Yonsei University College of Medicine, Seoul, Korea; 3 Graduate School of Yonsei University College of Medicine, Seoul, Korea

**Keywords:** Metabolic syndrome, Metabolic syndrome

## Abstract

This study investigated the endurance exercise-induced changes in lesser known adipokines (visfatin, chemerin, apelin, semaphorin 3 C) related to obesity and metabolism, and their correlations with the changes in the parameters of obesity and glucose homeostasis. Forty metabolically healthy obese young males were randomly assigned to control group (*C*, n = 12) or exercise group (*Ex*, n = 28). The subjects in *Ex* participated in a 8-week supervised endurance exercise training program, comprised of four sessions of treadmill running at 65–70% of VO_2max_ per week. **S**erum levels of visfatin, chemerin, apelin, and semaphorin 3 C were significantly decreased in *Ex*. At baseline, apelin and semaphorin 3 C appeared to be correlated with obesity measures, including body mass index, % total fat and trunk fat, and waist circumference. Exercise-induced changes in these obesity measures significantly correlated with the changes in chemerin and semaphorin 3 C. Basal chemerin, apelin and semaphorin 3 C correlated with glucose homeostasis parameters, including fasting plasma glucose, fasting plasma insulin, homeostasis model assessment of insulin resistance and β-cell function, and quantitative insulin-sensitivity check index to different extents. Furthermore, the changes in apelin and semaphorin 3 C well predicted the improvements in glycemic parameters. We suggest that semaphorin 3 C is a novel adipokine involved in pathophysiology of obesity and metabolism, and that it is a biomarker representing an exercise-induced improvement in metabolically healthy obese young males.

## Introduction

Obesity is a medical condition in which excess body fat accumulates to the extent that presents risks for chronic diseases, including Type 2 diabetes mellitus (T2DM) and cardiovascular diseases (CVDs)^1^. Although obesity is an important risk factor for various metabolic diseases, metabolic disturbances, such as insulin resistance (IR),  are not always presented in obese individuals^2^. For example, the prevalence of IR was relatively low and IR was not related to body fat mass, as evidenced by euglycemic insulin clamp results from 1,146 obese subjects^3^. This suggests that T2DM and CVDs are not simple consequences of fat depots, and there may be other factors altering metabolic profiles in obese individuals. Accumulated evidence suggests that the adipose tissue is an active endocrine organ that secretes more than 600 bioactive adipokines^4^, and related studies have elucidated underlying mechanisms and contribution of adipose tissues to the regulation of energy homeostasis. Specific adipokines, such as leptin and adiponectin, are reported to be involved in the etiology of metabolic diseases, and obesity leads to the dysregulation of adipokine secretion; therefore, adipokines may represent the link between obesity and energy homeostasis^5^.

In a step toward understanding the biological roles of adipose tissue and its clinical relevance with metabolism, a number of novel adipokines have been identified over the last decade. Among, *relatively*, recently identified adipokines, visfatin, chemerin, apelin, and semaphorin 3 C (SEMA3C) are reported to be associated with obesity and T2DM. Visfatin is an adipokine that originally was reported to play a key role in maintaining pancreatic β cell function through nicotinamide adenine dinucleotide (NAD) biosynthetic regulation^6^, and it was shown to enhance glucose-stimulated insulin secretion and expression of the genes associated with pancreatic β cell functions in mice^7^. Although controversies exist regarding the relationship between visfatin and glucose homeostasis in humans, several cross-sectional studies have confirmed that plasma visfatin level increases in subjects diagnosed with obesity and T2DM^8^. Chemerin is an adipocyte-derived peptide which has been reported to have auto/paracrine effects on adipose differentiation, as well as endocrine effects in metabolism^9^. It was suggested as a better measure for insulin sensitivity than HOMA-IR in an euglycemic insulin clamp study on males without metabolic syndrome^10^. Chemerin treatment induced glucose intolerance in obese diabetic mice^11^, and attenuated insulin stimulated glucose uptake in primary human skeletal muscle^12^ and 3T3-L1 adipocytes^13^; therefore, chemerin may be an important regulating factor of glucose homeostasis. In addition, circulating chemerin levels appeared to be closely related to the measures of diabetes and obesity^14^. Another lesser known adipokine, apelin, appeared to be linked to obesity and glucose homeostasis^15,16^. Apelin was also shown to have endocrine potency, as it increased glucose uptake both in human adipose tissue^17^ and in 3T3-L1 adipocytes^18^. These studies suggested that apelin may increase to compensate for the disturbed metabolic and hormonal *milieu* in obesity or diabetes. SEMA3C, which has been recognized for its roles in cancer biology, was recently identified as an adipokine that is expressed and secreted from white adipose tissue from transcriptome data and secretome profiles^19^. Although there was no significant metabolic effect in isolated human adipocytes, SEMA3C appeared to be significantly correlated with weight changes, fat-cell hypertrophy, adipose fibrosis, and whole body insulin resistance in humans^19^. However, evidence supporting SEMA3C to be an adipokine linked to metabolism is currently limited.

Exercise training is an effective tool for preventing and improving metabolic diseases. Considering that adipokines are dysregulated in obesity and exercise training has positive effects on impaired adipokine system and systemic metabolic homeostasis, beneficial effects of exercise are partially mediated through the changes in some adipokines^20^. Although the positive effects of exercise training on the changes in leptin and adiponectin and their relevance with glucose homeostasis have been reported, there may be many other adipokines that have not yet been investigated in this context^21^. In this study, we investigated the endurance exercise-induced changes in novel adipokines, including visfatin, chemerin, apelin, and SEMA3C, and their correlations with the changes in metabolic parameters in young obese Korean males.

## Results

### Effects of endurance exercise program on biochemical measures

**Figure 1:**
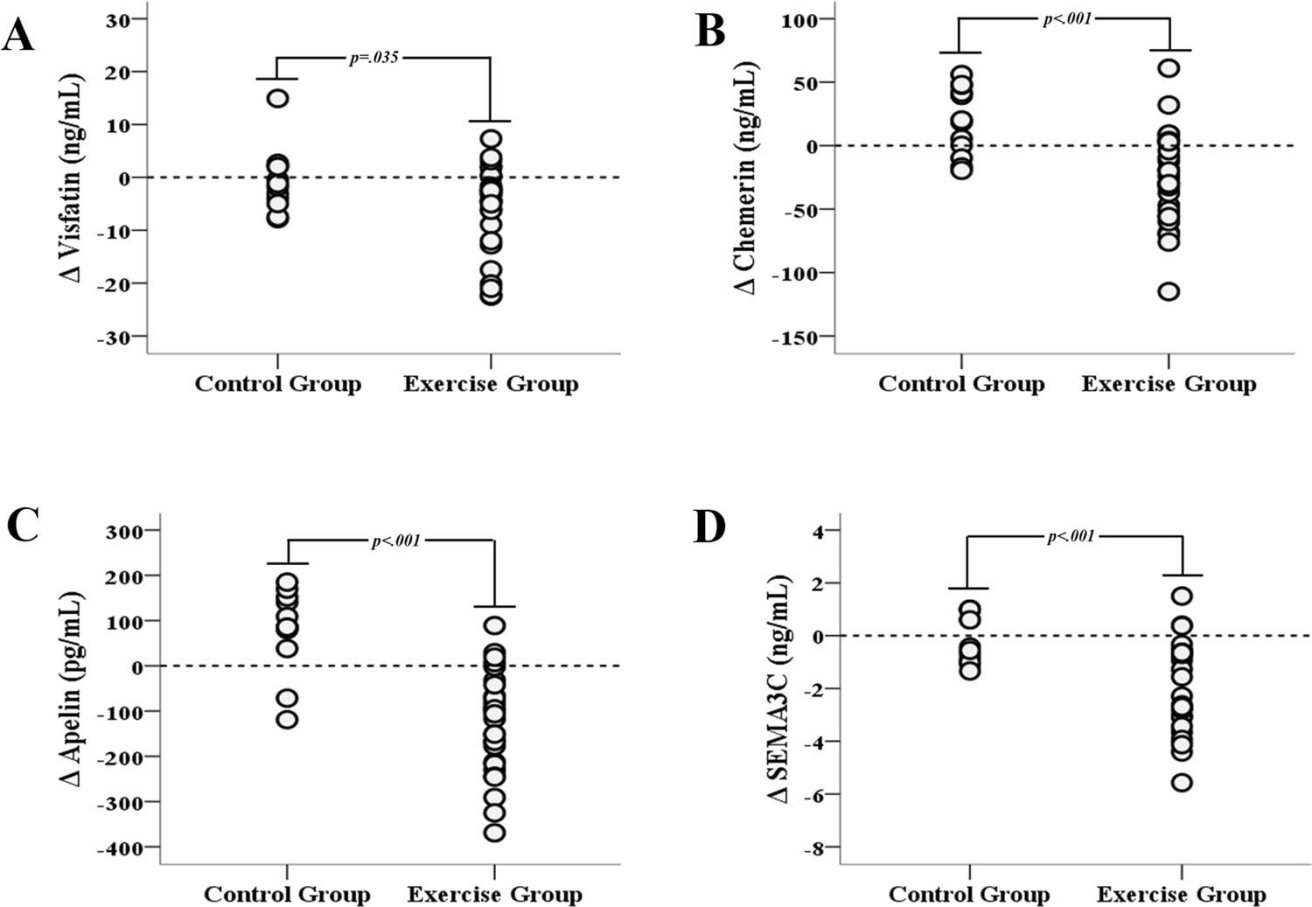
Changes in novel adipokines after 8 week-endurance exercise program. Statistical significant changes in serum levels of (**A**) visfatin, (**B**) chemerin, (**C**) apelin, and (**D**) SEMA3C in the exercise group compared to the control group were observed. SEMA3C, semaphorin 3C.

Clinical parameters at baseline and follow-up in *C* and *Ex* are shown in Table 1. *Ex* showed significant reductions in all obesity measures (BMI, % total fat, % trunk fat, and WC, all *p* < 0*.001*) and a significant increase in % lean body mass (*p* < 0*.001*) compared to *C*. FPI (*p* = 0*.005*), HOMA-IR (*p* = 0*.007*), and HOMA-β (*p* = 0*.039*) significantly decreased, while QUICKI (*p* < 0*.001*) significantly improved in *Ex*. LDL-C was significantly reduced in *Ex* (*p* = 0*.001*). In addition, *Ex* showed a significant decrease in systolic blood pressure (SBP) (*p* < 0*.001*). Diastolic blood pressure (DBP) in *C* significantly increased (*p* = 0*.041*), while *Ex* showed no change. Leptin (*p* < 0*.001*) was significantly reduced in *Ex*; while 8-week endurance exercise program decreased total adiponectin levels to near statistically significant level, it significantly increased the more biologically active high molecular weight (HMW) adiponectin^4^ in *Ex* (*p* < 0*.001*). The novel adipokines, visfatin (39.44 ± 13.41 to 33.48 ± 12.72 ng/mL, *p* = 0*.025*), chemerin (145.25 ± 66.31 to 118.14 ± 48.25 ng/mL p = 0.026), apelin (684.76 ± 198.22 to 555.29 ± 183.74 pg/mL*, p* = 0*.019*), and SEMA3C (6.95 ± 1.15 to 5.12 ± 1.95 ng/mL *p* = 0*.008*), significantly decreased in *Ex*. Statistically significant larger changes in these adipokines compared with *C* were shown. The changes are shown as individual delta values in Fig. 1

**Table 1:** Clinical Characteristics at Baseline and Follow-up.

	Control Group			Exercise Group				
	Baseline	Follow-Up	*p*	Baseline	Follow-Up	*p*	*p*^*a*^	*p*^*Δ*^
Age (yr)	27.08 ± 2.78	25.04 ± 3.01	0.050					
Weight (Kg)	85.94 ± 7.54	86.41 ± 8.37	0.341	89.59 ± 9.52	85.51 ± 9.07	<0.001^#^	0.388	<0.001
BMI	28.23 ± 2.28	28.40 ± 2.61	0.307	28.41 ± 2.37	27.14 ± 2.47	<0.001^#^	0.483	<0.001
Total Fat (%)	26.52 ± 3.21	26.59 ± 3.12	0.770	27.01 ± 2.95	24.39 ± 3.37	<0.001^#^	0.654	<0.001
Trunk Fat (%)	28.35 ± 3.89	29.26 ± 3.73	0.023^*^	28.91 ± 3.37	25.66 ± 6.07	<0.001^#^	0.945	<0.001
WC (*cm*)	97.62 ± 3.88	99.77 ± 5.04	0.010^*^	100.58 ± 4.96	91.79 ± 5.62	<0.001^#^	0.312	<0.001
FPG (*mg/dL*)	92.67 ± 7.66	93.83 ± 4.69	0.435	90.78 ± 5.29	88.26 ± 7.79	0.076	0.160	0.112
FPI (*uU/mL*)	4.20 ± 3.07	5.08 ± 3.11	0.285	4.66 ± 2.96	2.67 ± 1.87	<0.001^#^	0.998	0.005
HOMA-IR	0.97 ± 0.76	1.21 ± 0.80	0.266	1.14 ± 1.05	0.58 ± 0.44	<0.001^#^	0.681	0.007
QUICKI	0.40 ± 0.04	0.39 ± 0.04	0.194	0.39 ± 0.04	0.45 ± 0.03	<0.001^*^	0.580	0.001
HOMA-β (%)	50.29 ± 31.04	58.15 ± 29.05	0.330	60.09 ± 33.07	42.02 ± 25.40	0.004^#^	0.379	0.039
2h-AUC (*a.u*.)	14905.83 ± 2687.66	15212.92 ± 1943.32	0.194	15014.44 ± 1733.96	14560.37 ± 2052.68	0.120	0.254	0.103
TG (*mg/dL*)	129.1 ± 35.64	133.25 ± 46.56	0.662	97.22 ± 37.77	86.19 ± 46.79	0.171	0.715	0.260
TC (*mg/dL*)	180.58 ± 14.74	181.33 ± 31.92	0.930	174.93 ± 26.85	167.30 ± 24.24	0.076	0.065	0.318
HDL-C (*mg/dL*)	49.4 ± 8.24	49.67 ± 9.17	0.738	47.93 ± 7.63	50.59 ± 10.17	0.078	0.778	0.435
LDL-C (*mg/dL*)	103.92 ± 13.73	105.42 ± 25.58	0.852	109.44 ± 25.53	97.59 ± 21.94	0.001^#^	0.388	0.058
SBP (*mmHg*)	133.33 ± 9.75	132.17 ± 9.89	0.409	130.44 ± 10.01	125.81 ± 7.47	<0.001^#^	0.656	0.059
DBP (*mmHg*)	81.67 ± 7.98	83.83 ± 6.24	0.041^*^	84.89 ± 4.56	84.11 ± 3.66	0.094	0.008^*$*^	0.003
Leptin (ng/mL)	6.58 ± 2.83	6.66 ± 3.42	0.864	6.62 ± 2.65	2.68 ± 1.48	<0.001^#^	0.988	<0.001
TAdpn (ng/mL)	6.49 ± 2.80	5.80 ± 2.47	0.104	6.70 ± 2.66	6.03 ± 2.30	<0.001^#^	0.906	0.074
HMWAdpn (%)	2.68 ± 0.76	4.93 ± 5.99	0.207	2.31 ± 0.58	14.86 ± 8.53	<0.001^*^	0.079	<0.001

Values are mean ± SD; *significantly higher than pre-intervention within group; ^#^significantly lower than pre-intervention within group; ^*$*^significantly higher in *Ex*; ^a^*p* for pre-intervention measures between groups; ^*Δ*^*p* for the changes in measures between groups; BMI: body mass index; WC: waist circumference; FPG: fasting plasma glucose; FPI: fasting plasma insulin; HOMA-IR: homeostatic model assessment for insulin resistance; QUICKI: quantitative insulin sensitivity check index; HOMA-β: homeostatic model assessment for beta-cell function; 2h-AUG: area under curve from 2-hour oral glucose tolerance test; TG: triglyceride; TC: total cholesterol; HDL-C: high density lipoprotein cholesterol; LDL-C: low density lipoprotein cholesterol; SBP: systolic blood pressure; DBP: diastolic blood pressure; TAdpn; total adiponectin; HMWAdpm: high molecular weight adiponectin.

### Association between basal novel adipokines and other variables

Associations between the levels of novel adipokines at baseline and the parameters of anthropometry and glucose homeostasis in all subjects, regardless of groups*,* were examined (Table 2). Basal serum visfatin showed significant positive correlations with % trunk fat (*r* = 0*.355, p* = 0*.025*) and WC (*r* = 0*.480, p* = 0*.002*); serum apelin was positively correlated with BMI (*r* = 0*.454*, *p* = 0*.003*), % total fat (*r* = 0*.345, p* = 0*.029*), % trunk fat (*r* = 0*.422, p* = 0*.007*), and WC (*r* = 0*.353, p* = 0*.025*); and serum SEMA3C was positively correlated with BMI (r = 0.504, *p* < 0*.001*), % total fat (*r* = 0*.618, p* < 0*.001*), % trunk fat (*r* = 0*.684, p* < 0*.001*), and WC (*r* = 0*.451, p* < 0*.001*). However, serum chemerin did not demonstrate significant association with any anthropometric measures. Basal serum chemerin was correlated with FPI, HOMA-IR, and QUICKI (*r* = 0*.357, p* = 0*.024; r* = 0*.341, p* = 0*.031; r* = *−0.359, p* = 0*.023, respectively*); basal serum apelin was correlated with FPI, HOMA-IR, HOMA-β and QUICKI (*r* = 0*.672, p* < 0*.001; r* = 0*.603, p* < 0*.001; r* = 0*.696, p* < 0*.001; r* = *−0.613, p* < 0*.001, respectively*); and basal serum SEMA3C was correlated with FPG, FPI, HOMA-IR, HOMA-β and QUICKI (*r* = 0*.311, p* = 0*.044; r* = 0*.354, p* = 0*.028; r* = 0*.578, p* < 0*.001; r* = 0*.371, p* = 0*.019; r* = *−0.613, p* < 0*.001, respectively*). There was no significant association between basal serum visfatin and any glycemic parameters.

**Table 2:** Associations between Novel Adipokines and the Measures of Anthropometry, Glucose Homeostasis.

	Visfatin (37.58 ± 12.51 ng/mL)		Chemerin (141.83 ± 59.29 ng/mL)		Apelin (0.68 ± 0.19 ng/mL)		Semaphorin 3 C (7.19 ± 1.78 ng/mL)	
	*r*	*p*	*r*	*p*	*r*	*p*	*r*	*p*
***Anthropometric Measures***								
BMI	0.241	0.102	0.160	0.324	0.***454***	0.003^*^	0.***504***	***<***0.001^*^
%Total Fat	0.224	0.165	0.145	0.372	0.***345***	0.029^*^	0.***618***	***<***0.001^*^
% Trunk Fat	0.***355***	0.025^*^	0.167	0.304	0.***422***	0.007^*^	0.***684***	***<***0.001^*^
WC	0.***480***	0.002^*^	0.130	0.424	0.***353***	0.025^*^	0.***451***	***<***0.001^*^
% Lean Mass	0.187	0.401	−0.098	0.698	0.163	0.299	0.024	0.852
***Glucose Homeostasis Measures***								
FPG	0.263	0.121	0.167	0.317	0.044	0.788	0.***311***	0.044^*^
FPI	0.308	0.054	0.***357***	0.024^*^	0.***672***	***<***0.001^*^	0.***354***	0.028^*^
HOMA-IR	0.109	0.502	0.***341***	0.031^*^	0.***603***	***<***0.001^*^	0.***578***	***<***0.001^*^
HOMA-β	0.133	0.414	0.302	0.060	0.***696***	***<***0.001^*^	0.***371***	0.***019***
QUICKI	−0.033	0.842	***−***0.***359***	0.023^*^	***−***0.***613***	***<***0.001^*^	***-***0.***631***	***<***0.001^*^
2h-AUC	0.164	0.333	0.204	0.226	0.102	0.549	−0.310	0.051

Significantly associated with variables, *p *< 0*.05*. BMI: body mass index; WC: waist circumference; FPG: fasting plasma glucose; FPI: fasting plasma insulin; HOMA-IR: homeostatic model assessment for insulin resistance; 2h-AUG: area under curve from 2 hour oral glucose tolerance test.

### Associations between changes in novel adipokines and changes in other variables

Exercise-induced changes in the novel adipokines and the measures of anthropometry (Table 3) and glucose homeostasis (Table 4) were examined. The change in serum visfatin was significantly correlated with the changes in % trunk fat (*β* = 0*.478, p* = 0*.010*) and WC (*β* = 0*.381, p* = 0*.044*); the change in serum chemerin was significantly correlated with the changes in BMI (*β* = 0*.476, p* = 0*.010*), % total fat (*β* = 0*.689, p* < 0*.001*), % trunk fat (*β* = 0*.607, p* = 0*.001*), and WC (*β* = 0*.424, p* = 0*.025*); the change in serum apelin was correlated with only the changes in WC (*β* = 0*.419, p* = 0*.027*); and the change in serum SEMA3C was correlated with the changes in BMI (*β* = 0*.489, p* = 0*.009*), % total fat (*β* = 0*.535, p* = 0*.006*), % trunk fat (*β* = 0*.448, p* = 0*.018*), and WC (*β* = 0*.478, p* = 0*.010*).

**Table 3:** Correlation between the Changes in Anthropometric Measures and Novel Adipokines.

	Δ BMI		Δ %Total Fat		Δ %Trunk Fat		ΔWC		Δ % Lean Body Mass	
	*β*	*p*	*β*	*p*	*β*	*p*	*β*	*p*	*β*	*p*
Δ Visfatin	0.149	0.397	0.360	0.061	***0.478***	0.010^*^	***0.381***	0.044^*^	0.211	0.277
Δ Chemerin	***0.476***	0.010^*^	***0.689***	<0.001^*^	***0.607***	0.001^*^	***0.424***	0.025^*^	0.160	0.332
Δ Apelin	0.109	0.618	0.135	0.538	0.210	0.285	***0.419***	0.027^*^	0.367	0.057
Δ Semaphorin 3 C	***0.489***	0.009^*^	***0.535***	0.006^*^	***0.448***	0.018^*^	***0.478***	0.010^*^	0.150	0.384

*Significantly correlated among variables, *p* < 0*.05*. BMI: body mass index; WC: waist circumference.

**Table 4:** Correlation between the Changes in Novel Adipokines and the Glucose Homeostasis Measures.

	Δ Visfatin		Δ Chemerin		Δ Apelin		Δ Semaphorin 3 C	
	*β*	*p*	*β*	*p*	*β*	*p*	*β*	*p*
**No Adjustment**								
Δ FPG	***0.485***	0.009^*^	***0.484***	0.009^*^	0.098	0.621	***0.491***	0.006^*^
Δ FPI	0.167	0.396	0.075	0.706	***0.607***	0.001^*^	***0.611***	<0.001^*^
Δ HOMA-IR	0.182	0.353	0.096	0.627	***0.440***	0.019^*^	***0.543***	0.002^*^
Δ HOMA-β	−0.158	0.421	−0.313	0.105	***0.538***	0.003^*^	***0.370***	0.049^*^
Δ QUICKI	−0.344	0.073	−0.211	0.281	***−0.491***	0.008^*^	***−0.512***	0.006^*^
Δ AUC	−0.018	0.926	0.173	0.380	0.046	0.815	0.332	0.101
**Adjusted for Δ in BMI, Fat. Truncal Fat, WC, Lean Body Mass**								
Δ FPG	0.265	0.310	0.236	0.346	.−0.071	0.755	***0.405***	0.039^*^
Δ FPI	0.154	0.521	0.265	0.313	***0.542***	0.007^*^	***0.594***	0.001^*^
Δ HOMA-IR	0.130	0.537	0.293	0.260	***0.437***	0.036^*^	***0.534***	0.003^*^
Δ HOMA-β	0.094	0.711	−0.058	0.833	***0.612***	0.001^*^	***0.442***	0.019^*^
Δ QUICKI	−0.259	0.283	−0.346	0.129	***−0.525***	0.021^*^	***−0.435***	0.036^*^
Δ AUC	0.084	0.806	0.171	0.495	−0.203	0.311	0.176	0.381
**Adjusted for Δ in Leptin, Adiponectin, HMW Adiponectin**								
Δ FPG	0.287	0.270	***0.416***	0.038^*^	0.098	0.625	***0.488***	0.008^*^
Δ FPI	0.138	0.502	−0.058	0.783	***0.561***	0.002^*^	***0.530***	0.006^*^
Δ HOMA-IR	0.111	0.585	−0.008	0.967	***0.367***	0.049^*^	***0.451***	0.012^*^
Δ HOMA-β	−0.104	0.629	−0.380	0.071	***0.508***	0.010^*^	***0.397***	0.045^*^
Δ QUICKI	−0.322	0.107	−0.073	0.727	***−0.466***	0.014^*^	***−0.523***	0.005^*^
Δ AUC	0.052	0.796	0.312	0.119	−0.285	0.205	0.256	0.322

^*^Significantly correlated among variables. FPG: fasting plasma glucose; FPI: fasting plasma insulin; HOMA-IR: homeostatic model assessment for insulin resistance; HOMA-β: homeostatic model assessment for beta function; QUICKI: quantitative insulin sensitivity check index; 2h-AUG: area under curve from 2-hour oral glucose tolerance test; Ex: exercise group.

In simple linear regression, the changes in serum levels of visfatin and chemerin were positively associated with a change in FPG (*β* = 0*.485, p* = 0*.009; β* = 0*.484, p* = 0*.009, respectively*); these associations to the changes in FPG were still significant after adjusting for the changes in conventional adipokines. The change in serum apelin was positively correlated with the changes in FPI, HOMA-IR and HOMA-β (*β* = 0*.607, p* = 0*.001; β* = 0*.440, p* = 0*.019; β* = 0*.538, p* = 0*.003, respectively*) and was negatively correlated with QUICKI (*β* = *−0.491, p* = 0*.008*); these associations remained significant after adjustment for the changes in conventional adipokines and anthropometric measures. The change in serum SEMA3C was positively correlated with the changes in FPG, FPI and HOMA-IR (*β* = 0*.491, p* = 0*.006; β* = 0*.611, p* < 0*.001; β* = 0*.543, p* = 0*.002, respectively*) and was negatively correlated with QUICKI (*r* = *−0.512, p* = 0*.016*); these associations remained significant after the adjustments for the changes in conventional adipokines and anthropometric measures.

## Discussion

In this study, we demonstrated that our 8-week endurance exercise training program improved body compositions and metabolic parameters, and reduced serum levels of visfatin, chemerin, apelin, and SEMA3C. The changes in obesity parameters appeared to be correlated with the changes in serum chemerin and SEMA3C. In addition, we showed that, among four novel adipokines, exercise-induced change in serum SEMA3C independently predicted the improvements in parameters of glucose homeostasis. This is the first study to investigate the exercise training-induced change in SEMA3C and its relevance to glucose homeostasis.

Visfatin was identified as an adipokine based on the findings that it is mainly produced in adipose tissue and its serum level increases in parallel with visceral fat in both mice and humans^22^. Several clinical studies reported that serum visfatin level correlates with body fat mass, especially the abdominal and visceral fat^23,24^. In line with these, serum visfatin in our subjects at baseline appeared to be correlated with % trunk fat and WC, which are efficient predictors for visceral fat^25^. Our endurance exercise training program significantly reduced serum visfatin level, as previous studies did^24,26^; moreover, the changes in serum visfatin and % trunk fat and WC after the exercise training were significantly correlated in our subjects. These suggest that visfatin is a visceral fat-derived adipokine whose secretion is regulated by exercise-induced fat reduction. However, it has been suggested that serum visfatin level is not only determined by the extent of visceral fat, but can be stimulated by a hyperglycemia *milieu* such as Type 1 diabetes mellitus (T1DM) and T2DM^8^. Elevation of visfatin may be a physiological response to increase insulin secretion in hyperglycemic environment. To support this, Lopez *et al*. reported that circulating visfatin was inversely and independently associated with insulin secretion, rather than insulin sensitivity^27^. In addition, Brown *et al*. showed that visfatin regulates insulin secretion, insulin receptor signaling, and mRNA expression of β cell function-associated genes, such as Ins, HNFβ, and HNF4α, in murine β-cells^7^. Our study subjects, unlike other studies^8,23,24^ showed no significant association with the measures of glucose homeostasis; this discrepancy may have resulted from the normoglycemic status of our subjects. Although there was an association between the changes in serum visfatin and FPG, this association became non-significant after adjustment for the changes in anthropometric measures or serum levels of leptin, adiponectin, and HMW adiponectin, which are adipokines that have been reported to beneficially affect glucose metabolism^4,5^. In contrast to our finding, other studies reported that weight reduction by endurance exercise was associated with increased circulating visfatin and improved insulin sensitivity in obese diabetic patients^28^, and that exercise-induced change in visfatin appeared to be associated with the corresponding change in insulin sensitivity in old obese subjects^29^. These suggest that the effects of exercise on visfatin production and secretion may vary according to age and metabolic status of subjects, although serum visfatin levels did not exhibit significant correlations with gender^30^ and age^31^. Taken together, we suggest that visfatin is an adipokine whose secretion and serum level are regulated by exercise-induced visceral fat reduction. However, its implication in the exercise-mediated improvement in glucose metabolism is not clear in metabolically healthy obese individuals.

Chemerin was originally identified as a chemotactic agent that is presented in skin^32^, and now it has been added to the list of adipokines based on the reports that white adipose tissue highly expresses chemerin along its functional receptor, CNKLR1^9,33^. Current evidence suggests that chemerin plays a crucial role in adipogenesis, and this has been implicated in the control of adipose tissue regarding the regulation of glucose homeostasis and the development of obesity. Clinical studies have consistently reported that its gene expression and circulating levels are positively correlated with obesity parameters^12,33,34^. In support of this, high fat-induced obese mice^35^ and genetically obese *db/db* mice^11^ also showed increased circulating chemerin levels compared to chow control and C57BL/6 control, *respectively*; adipocytes isolated from obese individuals secreted chemerin significantly more than those of lean individuals^12^. Considering that chemerin was defined as an important regulator in adipocyte differentiation^9,36^, it can be suggested to be an adipokine involved in the pathogenesis of obesity. In our study, serum chemerin levels did not show significant positive correlations with any of the anthropometric measures, which may be due to the relatively narrow range of BMI and fat proportions across our subjects. However, serum chemerin level decreased after the training, as shown in previous studies with humans^34,37,38^ and mice^39,40^. The change in serum level also correlated with the changes in BMI, % total fat, % body fat, and WC. These suggest that chemerin is an adipokine whose secretion can be altered by exercise-induced adiposity reduction. Chemerin is generally elevated in T2DM patients^33,34^, especially in older T2DM patients^41^. Moreover, chemerin was suggested to be a better measure for insulin sensitivity than HOMA-IR in normoglycemic subjects^10^. Both glucose-stimulated insulin secretion from pancreatic β cells and insulin-stimulated glucose uptake in peripheral tissues are major features in the regulation of glucose tolerance. In this physiology, chemerin showed deteriorative effects on insulin sensitivity, while it enhanced insulin secretory response. For example, chemerin attenuated glucose uptake in skeletal muscle cells^12^, adipocytes^13^ and liver^11^. On the other hand, glucose stimulated insulin secretion in chemerin-knockout mice was attenuated^36,42^ while that in chemerin-transgenic mice was enhanced^42^. These suggest that elevated chemerin in those with glucose intolerance may be the compensatory consequence to stimulate insulin secretion. Significant positive associations with FPI and HOMA-IR and a significant negative association with the measure of insulin sensitivity (QUICKI) in our study also suggest that high serum chemerin is an unappreciated phenomenon for glucose homeostasis. Endurance exercise can reduce serum chemerin level, and this change may contribute to improved glycemic control^34,37,38^. Our endurance exercise program also significantly decreased serum chemerin level, and this change was significantly correlated with the change in FPG. This correlation remained significant after the adjustment for the changes in serum levels of leptin, adiponectin, and HMW adiponectin. Taken together, chemerin is an adipokine whose secretion is regulated by exercise induced fat reduction, and is somewhat associated with glycemic control in metabolically healthy obese individuals.

Apelin is a peptide that is expressed in multiple organs^43^ and has been recently identified as an adipokine, as its expression and secretion from humans and murine adipocytes have been confirmed^15^. Apelin, along with its receptor, AJP, is involved in a wide range of physiological functions, such as regulation of cardiac and vascular functions, fluid homeostasis and angiogenesis, by activating different G-proteins, depending on the cell type^43^. In addition, apelin–APJ system was reported to be associated with the pathophysiology of T2DM and obesity^44^. Apelin was shown to increase adipogenesis in primary human adipocytes^17^ and murine 3T3L1- adipocytes^18^. Both serum apelin and mRNA in adipose tissue appeared to be significantly correlated with BMI, % body fat, adipocyte size, and insulin sensitivity among 740 normoglycemic and diabetic subjects^45^. Other studies with small samples also reported higher serum apelin in obese diabetic individuals compared to lean normoglycemic controls^16,46–48^. Furthermore, apelin was suggested to be a biomarker that predicts T2DM incidence in men^49^. In line with these, serum apelin level, in our study, exhibited positive correlations with BMI, % total fat, % body fat, and WC, FPI, HOMA-IR, and HOMA-β, and a negative correlation with QUICKI. Our endurance exercise program significantly reduced serum apelin, as previous studies did^45,50,51^; this change, however, correlated only with the change in WC among anthropometric measures. We measured apelin-13, among its various fractions, which may represent the adipocyte-derived apelin as it is cleaved by subtilisin/kexin 3 (PCSK3), whose expression is increased in adipose tissue with obesity^52^, while others measured different fractions; therefore, further studies measuring the identical apelin fraction is warranted to investigate the effects of exercise on adipose tissue-derived apelin. Our results suggest that the changes in apelin by endurance exercise is not a simple consequence of adiposity-reduction, and that adiposity-reduction does not predict the change of apelin in metabolically healthy obese individuals. Exercise-induced change in apelin in our subjects appeared to be positively correlated with the changes in FPI, HOMA-IR, and HOMA-β, and negatively correlated with the change in QUICKI. These correlations still remained significant when anthropometric measures and classic adipokines were controlled. Insulin increased apelin expressions in both murine and human adipocytes, whereas the lack of insulin in T1DM mice showed decreased apelin expressions in their adipose tissues^15^. These suggest that apelin in adipocytes appeared to be directly regulated by insulin. Considering that increased insulin level is the most common phenomenon in obesity and in the state of insulin resistance^1^, elevated serum apelin in obese or insulin-resistant individuals may be due to the apelin-tropic effect of insulin on adipocytes. Decreased serum insulin level following our endurance exercise program was attributed to lower insulin demand that matches enhanced peripheral insulin sensitivity, and this decrease actually contributed to the changes in HOMA-IR, HOMA-β, and QUICKI. These suggest that apelin can be used as an exercise-induced biomarker representing insulin sensitivity in normoglycemic obese individuals. However, a previous study reported a significant increment in apelin serum concentrations after endurance exercise intervention among aged obese T2DM male and female Caucasians^53^. As serum apelin was not shown to be affected by gender and age^45^, more research is needed regarding this matter.

SEMA3C is a secreted protein initially discovered for its roles in the development of nervous, cardiorespiratory, and renal systems, as well as in different oncogenic processes^54^. Mejhert *et al*.^19^ has identified SEMA3C as an adipokine, based on the findings that it is primarily secreted from subcutaneous adipose tissues and its expression is regulated by the degree of obesity, fat-cell morphology, and weight changes. Moreover, mRNA expression of SEMA3C in adipose tissue was associated with insulin sensitivity, suggesting pathophysiological roles in human obesity and metabolic deterioration. In line with this, serum SEMA3C levels in our subjects appeared to be positively correlated with BMI, % total and trunk fat, WC, FPG, FPI, HOMA-IR, and HOMA-β, and negatively correlated with QUICKI. Our endurance exercise program significantly reduced serum SEMA3C, and this reduction was significantly associated with the changes in the associated parameters, even after the adjustments for obesity measures and classic adipokines. Although a previous study showed decreased SEMA3C expression in white adipose tissues after weight changes by calorie-restriction or bariatric surgery^19^, our study is the first to report that exercise-induced adiposity reduction resulted in decreased serum SEMA3C, which correlated with improved glycemic parameters. Although the physiological effects of SEMA3C via Plexins/Neuropilins has been investigated extensively in cancer biology^54^, its roles in obesity and glucose metabolism also has been suggested. SEMA3C was reported to bind, *specifically*, with Plexin D1^55,56^ which has been implicated in the development of abdominal obesity and T2DM through the regulation of ECM microenvironment^57^ and adipose tissue fibrosis^19^. Adipose fibrosis has been suggested as a hallmark of metabolically challenged adipocytes in diabetic state and is negatively associated with systematic metabolism^58^. SEM3C has been known to transactivate multiple receptor tyrosine kinases and downstreams such as phosphoinositide 3-kinase (PI3K)/protein kinase B (*AKT*) signaling pathway, which is causally associated with obesity and glucose metabolism by promoting lipid biosynthesis and glucose uptake and by inhibiting lipolysis^59^. Based on these, SEMA3C can be suggested as a potent molecule which participates in the pathophysiology in obesity and metabolism. Although it is not known whether attenuated adipose tissue fibrosis after endurance exercise training is attributed to reduced SEMA3C expression, we propose that SEMA3C is a novel adipokine which level is regulated by exercise-induced adiposity reduction and correlates with energy metabolism in healthy young obese males. However, more research with diverse subjects in different ages, gender and metabolic status is warranted to suggest SEMA3C as an adipokine representing exercise-induced improvements of obesity and metabolic impairment.

In this study, we comprehensively investigated the effects of 8-week endurance exercise training program on visfatin, chemerin, apelin, and SEMA3C and its relations to changes in the measures of obesity and glucose homeostasis in 40 obese young adults without metabolic derangements. Visfatin, chemerin, and apelin have been shown to be associated with obesity and glucose metabolism, their potentials to be used as novel biomarkers are somewhat inconclusive based on current evidence. Based on our results, SEMA3C seems to be a better serum marker to represent the exercise-induced improvements of metabolic status, as its change with endurance exercise training was well correlated with changes in obesity parameters and it better predicted the improvements of the parameters of glucose homeostasis compared to others. The limitation of our study is that we cannot determine whether the changes in these adipokines were due to decreased adiposity, exercise training or both. To elucidate this uncertainty, further studies which include both exercise and calorie-restriction groups are warranted.

## Methods

### Participants

Forty young sedentary males (mean age: 25.9 ± 2.3 yrs) who were diagnosed with obesity according to the criteria for Asians established by World Health Organization (WHO) and met the following criteria were included in the current study:1) subjects were weight-stable, with less than 5% of weight changing within 2 months before enrollment;2) subjects were sedentary and spent less than 20 minutes per day for continuous, regular physical activity in the past month, and never engaged in a structured exercise-training program before;3) subjects did not have any history of systemic diseases, infections, smoking, and medications for metabolic diseases;4) subjects were not physically disabled to undergo endurance exercise training program. Initially, 50 subjects were randomly assigned either to the control (*C*, *n* = 20) or the exercise (*Ex*, *n* = 30) group. Four subjects from *C* were excluded as they did not show up for follow-up examination, and four subjects from *C* and two subjects from *Ex* were further excluded, as some of their novel adipokine results lied out of the detection ranges of the assay kits. In detail, SEMA3C and chemerin levels of two subjects from *C* and one subject from *Ex* were below the detection ranges of the assay kits, and visfatin levels of two subjects from *C* and one subject from *Ex* were above the detection ranges of the assay kit; *accordingly*, data of 40 participants (12 in *C*, 28 in *Ex*) were finally analyzed in this study. Characteristics of the subjects in each group at baseline and follow-up are presented in Table 1. This work conformed to the standards set by the 64^th^ WMA Declaration of Helsinki, and the study protocol was approved by the Institutional Review Board of Yonsei University College of Medicine. All subjects provided their written informed consent before entering the study.

### Endurance exercise training program

Before the start of an 8-week supervised endurance exercise training program, cardiorespiratory fıtness (VO_2max_) of each subject was assessed by Bruce protocol^60^. Subjects participated four exercise sessions, which comprised of treadmill running at an intensity of 65–70% VO_2max_ to burn approximately 600 Kcal, per week under the provision of designated trainers. VO_2max_ of subjects in *Ex* were measured after every eight sessions to maintain the exercise intensity throughout the program. All subjects maintained their habitual recreational physical activities during the study period. Subjects in *C* maintained their normal lives.

### Anthropometric/blood pressure measurement

Body weight and height were measured with an electronic anthropometry measuring device (BSM370, BioSpace, Seoul, Korea) to the nearest 0.01 kg and 0.01 cm, *respectively*; and BMI was calculated as weight/height^2^ (kg/m^2^). Waist circumference (WC) was measured midway between the lowest rib and iliac crest in the standing position using a circumference measuring tape (SECA200, SECA, Hamburg, Germany). The percentages of total body fat, trunk fat, and lean body mass were measured by dual energy X-ray absorptiometry [(DXA); Hologic Delphi W, Bedford, MA, USA], and all DXA scans were analyzed with QDR software version 12.6. Blood pressure was measured by digital sphygmomanometer (EASY X800, Jawon Medical, Seoul, Korea) after 10-minute chair-rest.

### Biochemical analyses

All blood samples were collected after an overnight fast between 7:00 and 9:00 am and blood sampling for follow-up analyses were taken at least 48 hours after the last exercise session. Blood samples were immediately centrifuged at 3,000 *rpm* for 15 min at 4 °C, and the serums were collected and stored at −80 °C for subsequent analyses. Fasting plasma glucose (FPG), triglyceride (TG), total cholesterol (TC), high density lipoprotein cholesterol (HDL-C), and low density lipoprotein cholesterol (LDL-C) were quantified using an ADVIA 1650 Chemistry System (Siemens, Tarrytown, NY, USA). Fasting plasma insulin (FPI) was measured by ELECSYS2010 (Roche, Indianapolis, IN, USA). Whole body insulin resistance and insulin sensitivity were estimated by the homeostasis model assessment of insulin resistance (HOMA-IR) index [FPI (*uI*U/mL) x FPG (mg/dL])/405] and quantitative insulin sensitivity check index (QUICKI) [1/(log(fasting insulin, *uI*U/mL) + log(fasting glucose, *mg/dL*))], *respectively*. β-cell function was estimated by the homeostasis model assessment of β-cell function (HOMA- β) index [(360 × FPI (*uI*U/mL)/FPG (mg/dL) − 63]. During a standard 75 *g* oral glucose tolerance test (OGTT), blood samples were taken at 0, 30, 60, and 120 minutes after ingestion, and the product of blood glucose levels and time during OGTT were expressed as glucose area under the concentration time curve (AUC-Glu). The serum levels of leptin (RE53151, IBL International, Hamburg, Germany), total and high molecular weight (HMW) adiponectin (47ADPHU-E01, ALPCO, Salem, NH, USA), chemerin (ELH-Chemerin, RayBiotech, Norcross, GA, USA), apelin-13 and SEMA3C (MBS037239, MBS2883689, MyBioSource, San Diego, CA, USA) were measured by commercially available enzyme-linked immunosorbent assay (ELISA) assay kits. The serum level of visfatin was measured by commercially available enzyme immunoassay (EIA) kit (EK00380, Phoenix Pharmaceuticals, Burlingame, CA, USA). The detection ranges for leptin, total and HMW adiponection, chemerin, apelin-13, SEMA3C, and visfatin were 0.7~100 ng/mL, 0.075~4.8 ng/mL, 0.5~50 ng/mL, 1~100 ng/mL, 0.3~10 ng/mL, and 2.3~40 ng/mL, *respectively*. 1:5 dilution sera were used for total and HMW adiponection, and chemerin measurement while 1:2 dilution serum was used for SEMA3C measurement. The concentrations of the assayed samples were calculated by using 4-parameter curve fits based on the determined absorbance of the standards of each assay kit and the samples at 450 nm. The dilution factors were multiplied for total and HMW adiponection, chemerin, and SEMA3C for final determinations.

### Statistical analysis

All data were analyzed using Statistical Package for Social Sciences software (SPSS 22.0 K, IBM, Seoul, Korea), and are shown as mean ± SD. Baseline comparisons between the groups were assessed by independent sample *t-*test for the variables exhibiting normal distribution, and by Mann-Whitney *U* test for those not exhibiting normal distribution. To examine the differences between variables at baseline and follow-up within the group, paired *t-*test and Wilcoxin signed-rank test were performed, as appropriate. Pearson’s correlation analysis was used to evaluate the associations among baseline novel adipokines at baseline and the measures of anthropometry and glucose homeostasis. Simple linear regression and multiple linear regression analyses between the changes in the novel adipokines and the changes in the anthropometric measures, and multiple linear regression analysis between the changes in the novel adipokines and the changes in the measures of glucose homeostasis were performed to determine the relationship between the variables. In multiple linear regression analyses, the changes in glucose homeostasis parameters were used as dependent variables to determine the relative contribution of novel adipokines. To estimate the independent effect of each novel adipokines on the measures of glucose homeostasis, variables influence on glucose homeostasis, including anthropometric measures, leptin, total- and HMW adiponectin were adjusted. Levels of statistical significance were set at *p* < *0.05*.
